# Role of Endomyocardial Biopsy in Diagnostics of Myocarditis

**DOI:** 10.3390/diagnostics12092104

**Published:** 2022-08-30

**Authors:** Liga Vidusa, Oskars Kalejs, Aija Maca-Kaleja, Ilze Strumfa

**Affiliations:** 1 Department of Pathology, Riga Stradins University, 16 Dzirciema Street, LV-1007 Riga, Latvia; 2 Department of Internal Medicine, Riga Stradins University, 16 Dzirciema Street, LV-1007 Riga, Latvia; 3 Latvian Centre of Cardiology, Pauls Stradins Clinical University Hospital, 13 Pilsonu Street, LV-1002 Riga, Latvia

**Keywords:** myocarditis, inflammatory cardiomyopathy, endomyocardial biopsy, COVID-19

## Abstract

Endomyocardial biopsy as the cornerstone of diagnostics has been re-evaluated throughout the years, leaving unanswered questions on the precedence of it. The reported incidence of myocarditis has increased during the pandemic of coronavirus disease 2019 (COVID-19), reinforcing discussions on appropriate diagnostics of myocarditis. By analysis of evidence-based literature published within the last demi-decade, we aimed to summarize the most recent information in order to evaluate the current role of endomyocardial biopsy in diagnostics and management of myocarditis. For the most part, research published over the last five years showed ongoing uncertainty regarding the use, informativeness, safety and necessity of performing a biopsy. Special circumstances, such as fulminant clinical course or failure to respond to empirical treatment, were reconfirmed as justified indications, with a growing applicability of non-invasive diagnostic approaches for most other cases. We concluded that endomyocardial biopsy, if performed properly and with adjunct diagnostic methods, holds a critical role for treatment correction in specific histological subtypes of myocarditis and for differential diagnosis between immune-mediated myocarditis and secondary infections due to immunosuppressive treatment. A high level of possible misdiagnosing was detected, indicating the need to review terminology used to describe findings of myocardial inflammation that did not meet Dallas criteria.

## 1. Introduction

Myocarditis, defined by the International Society and Federation of Cardiology as inflammation of heart muscle based on specific histological criteria, has been estimated to affect more than a million patients annually. It can induce acute heart failure, ventricular arrhythmias and other life-threatening complications, historically resulting in high rates (6–10%) of mortality in young adults. In the long-term, myocarditis can lead to irreversible structural and functional changes, contributing for up to 30% of non-ischemic dilated cardiomyopathies [1,2,3]. Myocarditis can be triggered by a wide spectrum of etiological agents, ranging from infections and autoimmune diseases to cardiotoxic effects of various substances. Clinical manifestations are also diverse, from chest pain and dyspnea to cardiogenic shock of unknown origin as the first presentation. Initial findings within cardiac biomarkers, other laboratory tests, electrocardiography (ECG) and echocardiography (EchoCG) are mostly non-specific and can divert clinicians from considering an inflammatory process. Therefore, the diagnostic pathway towards suspected myocarditis begins with the exclusion of more common causes for the previously listed non-specific symptoms and findings. Angiographically significant coronary artery disease (CAD), valvular pathologies, congenital heart disease and extracardiac conditions that are able to induce heart failure symptoms have to be ruled out [2,3].

Cardiac magnetic resonance (CMR) is the radiological method of choice for further diagnostics, but findings are time-sensitive and can be non-specific as well. ^18^F-fluorodeoxyglucose Positron Emission Tomography/Computed Tomography (FDG PET/CT) can be used; however, different studies have yielded variable estimates of sensitivity and applicability. Other non-invasive approaches are still under investigation [2,3,4,5].

Endomyocardial biopsy (EMB) with the implementation of Dallas criteria, adjunct polymerase chain reaction (PCR) and immunohistochemistry (IHC) is considered to be the gold standard for definitive diagnosis, recommended for patients presenting with fulminant course or failure to respond to empirical treatment [2,3,4,5]. The role of biopsy for other cases remains unclear and has been the basis for discussion between professionals of the field for quite some time. Rapid technological progress allows to question the need for invasive diagnostic methods. However, the issue of diagnostics of myocarditis remains unresolved and has become increasingly topical in the midst of the rise in reported cases due to the pandemic of coronavirus disease 2019 (COVID-19). In addition, it has been estimated that statistical incidence of acute myocarditis could change with improvement of diagnostic procedures [6].

The aim of this review is to summarize the most recent data of the highest quality of evidence, in order to evaluate the current role of endomyocardial biopsy in diagnostics and management of myocarditis. 

## 2. Materials and Methods

**Figure 1:**
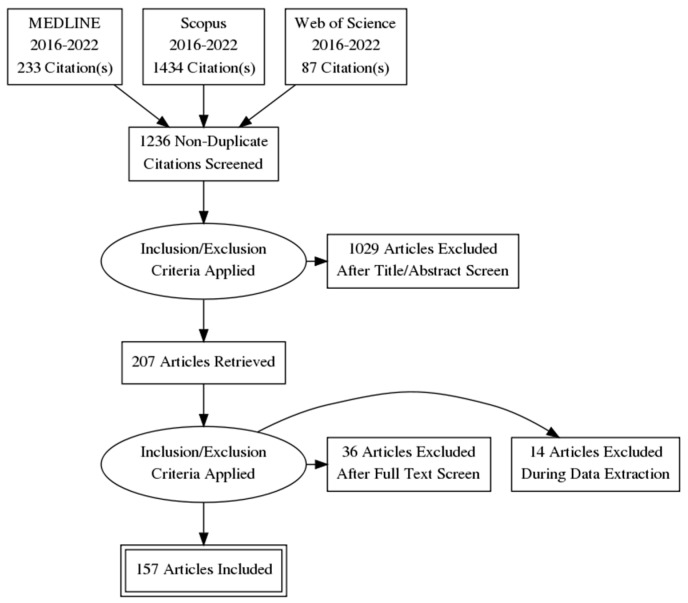
Flow diagram of literature search and selection using the PRISMA (Preferred Reporting Items for Systematic Reviews and Meta-Analyses) principle.

After the review question represented in the title was defined with the use of the PICO (problem-intervention-comparison-outcome) framework, the following terms and combinations of them were used to identify appropriate literature sources in PubMed, Scopus and Web of Science databases: “myocarditis”, “endomyocardial biopsy”, “inflammatory cardiomyopathy”. The article type filter of systematic review and review was applied, and publications of the last demi-decade were selected, setting the cut-off date to February of 2022. Identified studies were manually searched to uncover high-quality reviews. Selection criteria were determined as published systematic reviews and high-quality reviews, based on the Joanna Briggs Institute checklist, journal impact factor or cumulative citations, of any etiology myocarditis or inflammatory cardiomyopathy, containing information on the diagnostic approach and role of endomyocardial biopsy. Non-English, overlapping, inaccessible or reviews of low quality of evidence were excluded. Overall, 115 systematic reviews and 92 reviews were selected and analyzed to extract and summarize available data (Figure 1). Eleven systematic reviews and 39 reviews were excluded due to overlapping, accessibility and quality issues. Additional literature sources, such as guidelines and expert consensus statements were cited when necessary. Relevant statistical data, information regarding the diagnostic approach and findings for specific etiologies were collected and reflected in a concise manner.

## 3. Results

### 3.1. Definitive Diagnostic Methods of Myocarditis

Initial diagnostic assessment for patients with suspected myocarditis include detailed medical history, laboratory exams (including cardiac and muscular enzymes, liver and renal function, hemoglobin, differential white blood cell count, natriuretic peptides, thyroid function tests, iron status and screening markers of systemic auto-immune diseases), 12-lead ECG, ECG monitoring, EchoCG, invasive or CT coronary angiography. CMR imaging with T1 and T2 sequencing and LGE is indicated for all patients with symptoms in clinical history and detected cardiac biomarker, ECG or EchoCG abnormalities, with significant CAD excluded. Due to technological advancements, CMR has emerged as a noninvasive tool for tissue characterization, recognition and quantification of inflammation and fibrosis in patients with acute myocarditis or chronic inflammatory cardiomyopathy. Although EMB is the golden standard, it is recommended as an additional diagnostic test, for definitive diagnosis and treatment corrections in specific occasions [1,3].

#### 3.1.1. Cardiac Magnetic Resonance

Cardiac magnetic resonance, performed within a month of initial symptom presentation, represents the radiological method of choice if myocarditis has been suspected by clinical symptoms, elevated cardiac biomarkers, EchoCG findings and other common causes of these pathological changes have been excluded. The use of updated Lake Louise criteria is mandatory (Table 1). To reach the full informativeness of CMR, the time window of one month should be observed, except for patients in a critical general condition in whom CMR can be delayed [1,2,5].

Features suggesting active myocarditis upon CMR include T2-based imaging of myocardial edema and increased relaxation time, T1-based imaging of regional vasodilatation and increased extracellular volume, as well as non-ischemic necrosis with subsequent fibrosis shown by late gadolinium enhancement (LGE), supported by timing of clinical symptoms, pericardial findings and left ventricular wall motion abnormalities [5,7]. 

T1 and T2 CMR mapping of increased extracellular volume offers a comparably high diagnostic level for detection and accurate quantification of edema and fibrosis, seen in different stages of acute myocarditis. However, pathologies with similar presentation, previous myocardial damage or presence of chronic myocarditis can cause misdiagnosing with isolated use of CMR, as specificity reportedly declines in patients with a subsequent EMB performed [8,9,10,11]. Still, there is a good correlation between CMR and EMB findings, with up to 78% overall accuracy when updated Lake Louise criteria are applied [4,12,13].

In addition, non-ischemic LGE has been evaluated for prognostic purposes. LGE highlights early necrosis and fibrosis. Both of these processes interfere with functionality of the muscle and can serve as arrhythmogenic substrate, resulting in life-threatening complications. Reports highlight LGE burden and anteroseptal location as important prognostic markers for adverse cardiac events in patients with acute myocarditis, independently from age, gender, medical history, symptom severity, ECG and EchoCG findings or cardiac biomarker levels. Furthermore, patients surviving acute myocarditis have an 11.5% overall incidence of mortality from adverse cardiac events during 2-year follow-up, even with correction of left ventricular systolic function, offering findings of LGE as a possible predictive measure, as no others have been found so far [14,15,16]. FDG PET/CT can reveal the inflammation-related increase of metabolic activity; therefore, it can be used to replace CMR, complement it or monitor disease activity and progression during follow-up [5,7].

#### 3.1.2. Endomyocardial Biopsy

The indications for invasive diagnostics via EMB are set on the basis of pre-existing investigations and the likelihood of findings plausibly affecting treatment options or outcome. Higher class recommendations are focused on a recent onset of severe or fulminant heart failure and cases of ineffective empirical treatment, additionally considering the time criterion and clinical symptoms for the presumed etiological process (Table 2). EMB allows to evaluate histological composition of inflammatory cell infiltration, cardiomyocyte damage with necrosis, fibrosis, atrophy or hypertrophy of muscle fibers and specific findings, which include eosinophils, granulomas, giant multinucleated cells and pathognomonic findings indicative of etiological factors. PCR is added for pathogen detection, and IHC—for inflammatory infiltrate analysis. 

The definitive diagnosis of acute myocarditis is based on Dallas criteria for active myocarditis—myocardial infiltration with mainly mononuclear cells, signs of necrosis and non-ischemic myocyte degeneration. Borderline findings are defined as sparse infiltration without myocyte injury. Updated Dallas criteria (Table 3) by the position statement of European Society of Cardiology in 2013 are stated as the infiltrate containing ≥14 leukocytes/mm^2^, including up to 4 monocytes/mm^2^ and cluster of differentiation (CD) 3+ T-lymphocytes ≥ 7 cells/mm^2^ [1,2].

Unfortunately, EMB is subjected to problematic issues. Acquiring a biopsy from inflamed sites can be difficult due to their patchy nature or anatomic inaccessibility. Dallas criteria are known to have low sensitivity (60%) and high subjectivity (up to 64%), leading to low interobserver agreement between pathologists. Either positive or negative PCR findings in regard to the presence of an infectious agent within the myocardium can have unknown significance. IHC results may vary in patients with the same etiology of myocarditis [1,4,5].

To increase the diagnostic quality, transthoracic EchoCG with two-dimensional speckle tracking is helpful to select patients within early stages of myocardial involvement, especially among patients affected by pre-existing conditions that can increase the likelihood of subclinical inflammation [17,18]. The diagnostic yield is higher when EMB is performed within the first two weeks of the onset of clinical symptoms. Risk of complications is lower with EchoCG guidance and if hemodynamic stability has been achieved at least 24 h before [19,20]. At least five separate sample sites have been suggested to improve biopsy informativeness and correspondence with Dallas criteria, keeping in mind that autopsy studies have shown the criteria lacking sensitivity not only due to the inflammatory process being spatially heterogeneous but transient as well, with occasionally fast development of fibrosis, making it difficult to distinguish its genesis histologically from other possible causes [21]. A synergy of ECG and imaging methods such as EchoCG, CMR and electroanatomic mapping is advised for the best guidance of higher quality biopsy sampling [20,22]. 

Still, as field professionals have stated, reasons for not performing an EMB are mainly related to safety concerns, not the lack of its informativeness. Complication risk for both right and left-sided biopsies, when performed by experienced professionals is known to be low (<1%). Studies have evaluated trans-radial access and the use of imaging guides for an even further risk reduction, as the most common adverse event for adults is a hematoma at the access site. Scarce data are available regarding EMB in children, with recent reports showing an overall higher complication rate (15.5%), especially in infants and those weighing under 8 kg [4,17,19].

### 3.2. Viral Myocarditis

Viral infections are the most frequently reported etiological factors of acute myocarditis, presumed even in cases where the viral pathogen cannot be detected, or when it could act as a trigger for a secondary reaction. No consensus exists regarding the necessary investigations to confirm infectious causes. Coronary artery disease and congenital or acquired structural deformities are routinely investigated, but dysfunction induced by toxic substances, collagen vascular diseases, postpartum cardiomyopathy and myocardial infarction with non-obstructed coronary arteries are just a few of the examples that occasionally can cause misdiagnosis, especially in patients having a coincidental history of a recent viral illness. The COVID-19 pandemic brought attention to viral myocarditis and problems with its diagnostics. To solve the listed controversies, increased awareness about the informativity of EMB is necessary, based on the understanding of the pathogenetic pathways [4,21,23,24].

#### 3.2.1. Viral Myocarditis of Unspecified Etiology

There are some general patterns in the pathogenesis of viral myocarditis that have been identified in biopsy and autopsy studies. The early damage classically is caused by direct viral activity and replication inducing myocyte necrosis, release of intracellular contents and a maladaptive immune response, which includes enhanced synthesis of interleukin-6 and other cytokines, autoantibody production or direct inflammatory myocardial infiltration. In other cases, molecular mimicry between cardiomyocyte surface receptors and pathogens has been described. Natural killer cells and macrophages are known as first responders, followed by T-lymphocyte infiltration, causing inflammation that lasts weeks or even months and may result in either full remission or residual structural changes. Cardioprotection can be achieved with knowledge of these processes and reduction of their effects on the inflammatory stage, which can be detected early on, using a non-invasive approach, e.g., EchoCG and CMR. Still, in order to gain knowledge of cellular reactions, tissue sample acquiree and analysis is needed [25,26]. 

Not all viruses follow the same pattern of infection. Enteroviruses, adenoviruses and Zika virus are able to infect cardiomyocytes directly, parvovirus B19 can cause latent endothelial infection but Dengue virus presents with acute endothelial dysfunction. Influenza and coronaviruses can induce an immediate immune-mediated response, but human herpesvirus type 6, cytomegalovirus and Epstein–Barr virus are found in the majority of the population in latent forms and can reactivate in immunocompromised patients [23,27,28,29]. Therefore, the need for use of unified terminology has been actualized, distinguishing between virus-mediated and virus-triggered myocarditis and EMB with an adjunct PCR can help to distinguish between them [1]. 

Viral serology and positive peripheral blood PCR findings have not shown to be useful in precisely detecting the causative pathogen. However, the presence or absence of the pathogen in EMB and peripheral blood can help to evaluate the activity of a systemic infection or possibility of an endogenous reactivation [1,2,30]. Frequently, multiple infectious agents of unknown significance are found by PCR of EMB samples. It is believed that findings of a low active pathogen concentration in biopsy materials could be caused by delayed EMB acquiree within the chronic phase, inaccurate choice of ventricle, low sample quantity and sampling errors. In cases of low pathogen presence, a high value of perforin-positive infiltrates (>2.95 cells/mm^2^) has been proposed as a predictor of expectant deterioration of left ventricular ejection fraction [4,21,31]. 

To differentiate viral myocarditis from immune-mediated myocarditis in absence of the detected viral pathogen within EMB, several cardiac autoantibodies (e.g., anti-myosin, anti-fibrillary, anti-intercalated disk antibodies) have been described as possible serum markers for an immune-mediated process [4,23,31,32,33]. Circulating microRNAs have also been proposed as differential diagnostic markers (e.g., hsa-miR-Chr8:96), still needing further research for confirmation [17,34]. 

Understanding of pathophysiological mechanisms, medical history data, infection markers and viral load measurements could alleviate or even exclude the need for invasive detection of pathogen presence within the myocardium for treatment choice. Reviews of immunosuppressive therapy in myocarditis noted that previous trials showed a neutral effect upon outcomes in patients with myocarditis of unspecified etiology and a positive effect upon patients with biopsy-proven myocarditis with virus-negative PCR findings, coinciding with recommendations in the expert consensus from the European Society of Cardiology that empirical treatment can be used when deemed necessary [2,6,35]. 

As majority of pediatric cases are caused by viral infections, EMB has been used as the basis for treatment corrections—the addition of antiviral therapy or restrictions of immunosuppression. Population studies showed that children with virus-positive PCR findings and limited immunosuppression had fewer adverse events than those that were treated with immunosuppressive therapy [19,36]. Conflicting results on immunosuppression benefit have been reported in studies of clinically suspected myocarditis in children, with evidence of overall treatment benefit without the knowledge of viral particle presence [37,38]. In contrast, PCR findings did not change prognosis in the adult population and virus-negative patients had better results from conventional heart failure treatment alone [39]. 

A high overall rate (60–70%) of complete recovery after viral myocarditis has been reported, with healed myocardium in control biopsies and a significant reduction of residual fibrosis or calcification from use of conventional heart failure therapy. However, severe and persistent infections, especially by enteroviruses, represent an exception, causing a 30% mortality rate in neonates and 25% 2-year mortality in adults. In such cases, EMB would be initially indicated because of the severe clinical course and can bring clinical benefit due to the addition of antiviral agents against enteroviruses [15,21,40]. 

Previous studies have confirmed the lack of correlation between the degree of inflammation and risk of arrhythmias or sudden cardiac arrest. The risk of rhythm disturbances is elevated in physically active young adults, due to exercise-caused aggravation of subclinical inflammation. This correlation emphasizes the need for activity restrictions and screening of athletes or otherwise highly active patients after viral infections that are known to have high cardiac tropism. ECG, EchoCG, Holter monitoring and CMR should be used for screening. Suggestive EchoCG findings include increased wall thickness, global left ventricular dysfunction, localized wall motion abnormalities and pericardial effusion. CMR can detect capillary hyperemia, myocardial edema and signs of myocyte injury. Exercise intolerance after cardiotropic infections, with tests performed under medical surveillance, could be used to select patients for further non-invasive investigations and EMB for ambiguous cases [41,42,43].

#### 3.2.2. COVID-19-Associated Myocarditis

The epidemiological importance of COVID-19 cannot be overestimated. Nevertheless, SARS-CoV-2 infection is notable also for the controversial issues that have been highlighted regarding the diagnostic criteria of myocarditis. 

**Figure 2:**
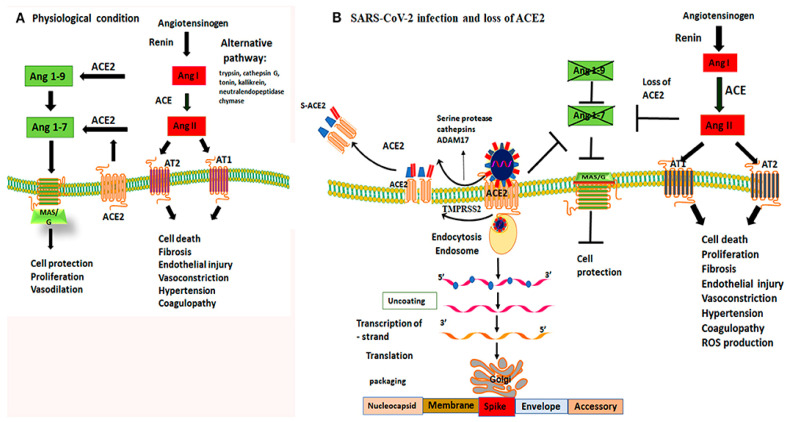
Mechanism of ACE2 receptor within physiological processes and COVID-19 infection. Figure replicated from [46] under Creative Commons license, provided at https://creativecommons.org/licenses/by-nc/4.0/ (accessed on 1 February 2022). Changes made: Figure legend. (**A**) Physiological conditions permit a balanced regulation of renin–angiotensin–aldosterone-mediated effects by the activity of both angiotensin-converting enzymes; (**B**) By binding to ACE2 receptors, the virus not only gains a point of entry, but is able to disrupt their protective capabilities, causing unbalanced activity of angiotensin I.

SARS-CoV-2 infection can cause serious and irreversible damage to multiple organs due to diffuse expression of angiotensin converting enzyme 2 (ACE2) receptor to which the virus binds, inducing direct cellular damage, dysregulation of the renin-angiotensin-aldosterone system and microangiopathy (Figure 2). The resulting tissue damage is caused by a systemic inflammatory response and demand–supply mismatch [44,45].

Clinically, symptoms of cardiac injury were seen in about a third of SARS-CoV-2 infected patients, correlating with higher mortality rates [44,47,48]. According to post-mortem assessment, the infection can induce myocyte ferroptosis and inflammatory infiltrates, edema in stroma and vascular walls, atrophy of cardiac muscle fibers, cardiac dilatation and focal necrosis or fibrosis, thus causing acute heart failure, reported as the second most frequent cause of mortality in SARS-CoV-2 infected patients. Although acute heart failure in some COVID-19 patients is attributable to direct cardiac injury, more frequently it occurred secondary to pulmonary overload, diffuse alveolar damage being the dominant pathological finding [49,50,51]. Initial screening for direct cardiac injury includes myocardial biomarkers, ECG and EchoCG. Interpretation of findings is difficult due to frequent cardiovascular comorbidities and the severe general condition of patients exhibiting cardiac symptoms. In autopsy samples, cardiac involvement was considered direct on the basis of specific histological findings, including infiltration of CD68+ macrophages and CD3+, CD4+ or CD8+ lymphocytes. The inflammatory infiltrate likely developed as a response to high levels of circulatory cytokines, such as interleukin-6 and tumor necrosis factor α [44,47,49]. 

Comparably lower susceptibility to COVID-19-associated myocarditis has been seen in children. The age-related pathogenetic differences confirm the importance of ACE2 receptor density, which is higher in adults and in patients with cardiovascular comorbidities. The immune response also differs by age—cytokine production intensity is lower and innate response adaptability is higher in children [50]. 

In a significant fraction (up to 48%) of SARS-CoV-2 infection-associated myocarditis, the cardiac diagnosis was confirmed only by EchoCG. Adjunct CMR was performed in only up to a half of those cases [52,53,54,55]. In patients who underwent CMR, non-ischemic LGE was found in less than a half (43%) of them, suggesting a reasonable likelihood of diagnostic errors [56]. An isolated rise in cardiac troponin levels could be assessed as a criterion for patient selection for CMR and EMB if elevated troponin levels are found in absence of other parameters, suggesting a severe course of the infection. This approach would be practical because cardiac troponins are routinely tested in COVID-19 patients as one of the predictors of outcome [57,58,59,60]. 

Overall, in reports of COVID-19-associated myocarditis, confirmation by EMB is neither frequent nor yields classical morphology. For example, in a single review, EMB was performed only in 20% of reported cases. The use of the procedure was likely limited by its invasive nature, general condition of the patient, unclear impact on further therapeutic decisions, concerns about infection control and shortage of medical resources during the pandemic. Regarding EMB findings, Dallas criteria were met only in a few of those cases. There was a high number of borderline findings with T-lymphocyte and macrophage infiltration and limited necrosis, highlighting the hypothesis of a virus-triggered hyperinflammatory response. Autopsy findings revealed diffuse inflammatory cell infiltration and varying signs of necrosis or ferroptosis; causing difficulties in distinguishing between direct involvement versus cardiac injury as a component of multiorgan failure in a severe course of the infection [57,61,62]. Cardiac inflammation was confirmed in 17.6% of overall samples (33.4% of biopsy specimens and 66.6% of autopsy materials) and only 11.4% of cases, showing inflammation, met the criteria for myocarditis. Dominant findings included thromboembolic events and endothelial inflammation with microvascular thrombosis. Thus, hypercoagulation and a hyperinflammatory response have a higher prevalence [63,64,65,66,67,68]. Other reviews mentioned signs of cardiac injury caused by exacerbations of preexisting conditions resulting in myocardial ischemia [64,66]. The role of hypoperfusion-related cardiac injury was supported by a review of coronary artery calcium score impact on mortality rates in COVID-19 patients, finding a two-fold increase in patients with a higher score [69]. Pediatric reviews showed a high incidence of clinically suspected myocarditis in children presenting with multisystem inflammatory syndrome [70,71,72,73]. There were only a few reported pediatric cases supported by CMR findings or confirmation by biopsy or autopsy [50,74,75]. The COVID-19 pandemic has also highlighted the need for EMB and autopsy sample collection to determine the correct underlying process and possible treatment directions, when facing novel pathogens [76,77].

Viral presence of SARS-Cov2 in EMB or myocardium autopsy samples tested with PCR was found in about a third (33%) of affected patients. Difficulties to determine the significance of these findings and distinguish between subcellular structures and viral particles were noted. It has been presumed that phagocyte migration from lung tissue could be a possible reason for a non-specific presence of viral particles [63,64]. In several cases, patients tested negative with nasopharyngeal swabs, but viral particles were found in postmortem cardiac tissue samples, suggesting that cardiac involvement could persist long after respiratory symptoms have resolved [57]. Even when high viral replication was reported (47% of autopsy samples), lymphocytic infiltration was scarce (approximately 10%), and myocarditis meeting Dallas criteria was confirmed only in 1.5% of cases. Detected viral presence was contributing to a combination of non-specific myocardial edema and endothelial inflammation, with dominant pathological changes in autopsy materials being cardiac dilatation, ischemia, intracardiac thrombi and pericardial effusion. These findings confirmed that cardiac injury mainly developed as a part of respiratory overload or multiorgan damage caused by hypoperfusion and hypercoagulation. Predisposition to this type of damage was observed in patients with preexisting cardiac conditions, such as myocardial hypertrophy or fibrosis [61,64].

CMR follow-up studies have shown that nonspecific myocardial edema as a muted response to direct viral activity tends to persist, possibly causing delayed or long-term implications [41]. CMR follow-up after 6 months yielded high frequency (46.5%) of pathological findings, with predominantly (87.9%) non-ischemic LGE pattern and T2 abnormalities, found in approximately a fifth of cases (20.7%). Persistent CMR abnormalities have been reported even in patients having normal cardiac biomarker levels on follow-up and lacking medical history of cardiac disease. LGE with T2-based criteria indicates myocarditis in the inflammatory phase. Non-ischemic LGE without T2 abnormalities indicates residual myocardial scarring and is associated with development of heart failure and arrhythmias after recovery. Thus, myocardial inflammation can persist long after clinical recovery from the infection. Prolonged follow-up studies and more frequent EMB acquirees are desired to determine the specifics of this persistent inflammation and its possible consequences. The extent of LGE can diminish significantly, as previous studies have shown at 12 months of follow-up [56,78,79,80,81,82]. 

COVID-19 vaccine safety reviews in children and adolescents disclosed myopericarditis at an estimated incidence of 0.01% in the age group between 12–15 years and 0.008% in 16–17 years old. Most of these cases exhibited a mild course [83]. In accordance with the latest population-based studies, young males were affected more frequently, usually a few days following their second dose, and the presentation was mainly self-limiting. It should be noted that CMR and EMB were rarely carried out, determining whether this reaction to vaccines meets any myocarditis criteria [17,84,85,86,87]. Autopsies have been performed in rarely occurring (38) fatal cases in whom the causality relationship was described as possible. The histological picture in these cases differed from viral or immune-mediated myocarditis: neutrophils and histiocytes predominated in the infiltrates [88].

Overall, literature on viral myocarditis suggests that knowledge of pathophysiological patterns holds high value over clinical choices. Medical history, infection markers and early detection of myocardial inflammation via non-invasive investigations, such as EchoCG and CMR, can guide the treatment plan, but knowledge of the inflammation pattern can increase its effectivity and prevent unnecessary actions. Without a sufficient number of tissue samples confirming similarities or specific nuances of different etiology viral myocarditis, this knowledge cannot be obtained [6,26,35,76,77]. 

### 3.3. Systemic Immune-Mediated Disease-Associated Myocarditis 

Statistical incidence of systemic immune-mediated myocarditis remains unknown, as the exact etiology often remains undetermined. Sarcoidosis, systemic lupus erythematosus, systemic sclerosis and disorders presenting with hypereosinophilic syndrome represent increasingly recognized diseases that can cause myocarditis [2,20].

**Figure 3:**
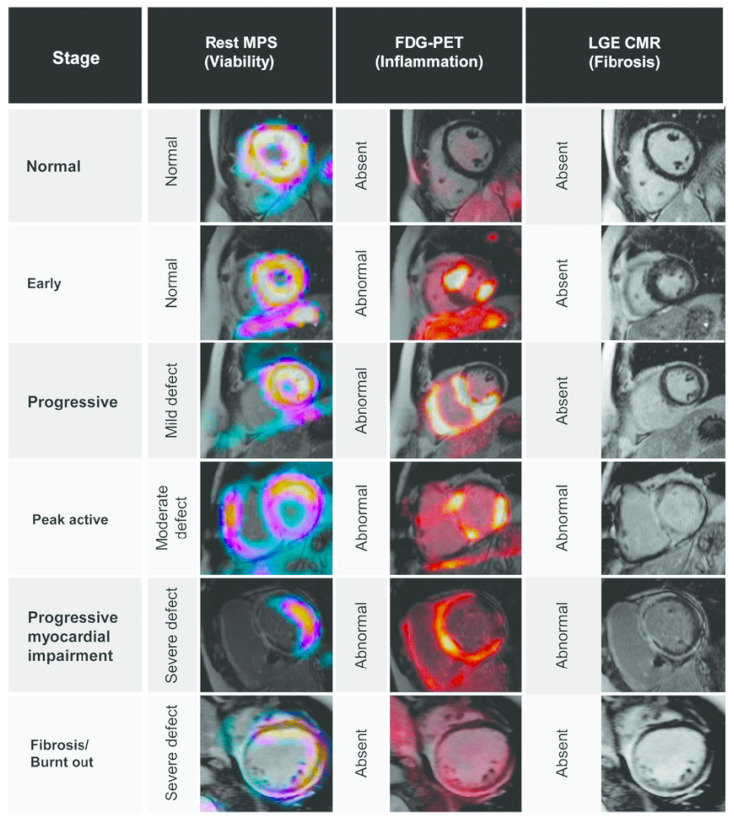
Use of FDG PET for early detection of cardiac sarcoidosis, showing FDG advantages in the ability to detect the activity of inflammation early on, in comparison with resting myocardial perfusion scintigraphy (MPS) and CMR. Figure replicated from [95] under Creative Commons license, provided at https://creativecommons.org/licenses/by-nc/4.0/ (accessed on 1 February 2022). Changes made: Figure legend.

Sarcoidosis is a systemic inflammatory disease of unknown etiology, characterized by non-caseating granulomas. Cardiac sarcoidosis has a considerably high reported frequency (20–78% of autopsy samples, depending on geographical region) and low recognition within clinical studies (less than 7%), due to the clinically silent course of cardiac lesions. In patients with extracardiac sarcoidosis, cardiac involvement was frequently found (up to 55%) by performing CMR. LGE upon CMR was observed in approximately one-fifth (19%) of patients in a large observational study of patients with extra cardiac sarcoidosis, lacking cardiac symptoms and featuring a preserved left ventricular ejection fraction. Serological markers such as anti-heart autoantibodies (AHA) and anti-intercalated disk autoantibodies (AIDA) show promise for patient selection, as they are found only upon cardiac involvement. Starting the treatment even in cases of suspicion and before systolic dysfunction can be detected by an EchoCG is known to ensure an improved clinical outcome, independently of other parameters [20,89,90]. Even though EMB remains the golden diagnostic standard to confirm cardiac involvement, its performance is rarely discussed in studies, with reports of low sensitivity (20–30%, due to the patchy distribution of the process), in comparison to non-invasive methods. A high number of cases (25%) have been confirmed only upon autopsy findings of granulomas and severe fibrosis. FDG PET/CT has a reported sensitivity of 89% and specificity of 83% for the diagnostics of cardiac sarcoidosis. PET/CT has been successfully used to visualize enhanced focal metabolism within the myocardium (Figure 3) and mediastinal lymph nodes, therefore increasing possibilities for myocardial involvement screening in patients with extracardiac lesions, specifying biopsy sites in isolated heart involvement and follow-up strategies for all groups. Issues related to physiologic uptake of FDG represent the main factor for inconclusive findings, therefore a synergy with other methods, such as CMR, would be optimal [20,30,91,92,93,94].

Systemic lupus erythematosus features reported primary heart involvement in up to 15% of cases. The median onset of myocarditis in patients with primary extracardiac manifestations has been estimated at 8.5 years after the diagnosis. The risk increases by overall disease activity, therefore, the Systemic Lupus Erythematosus Disease Activity Index can be used as a tool for patient selection for a targeted diagnostic approach. Lupus myocarditis is the most common (31%) rheumatological condition associated with cardiogenic shock. On the other hand, subclinical lupus myocarditis rarely progresses into clinical forms and has little prognostic significance. EMB remains the golden standard, especially for differential diagnosis between lupus myocarditis versus secondary myocarditis in patients receiving immunosuppressive treatment. Non-invasive investigations, such as CMR and PET, are also presumed to be sufficiently informative [20,91,96].

In systemic sclerosis presenting with internal organ involvement, cardiac pathology is one of the major predictors of mortality. Targeted cardiac screening in patients with known internal organ fibrosis or diffuse scleroderma should be performed, starting with EchoCG, optional serology of AHA and AIDA, CMR for detection of subclinical or early-stage disease and a definitive diagnosis with EMB if necessary. Myocardial fibrosis in these patients can be immune-mediated or caused by Raynaud’s phenomenon and repeated coronary vasospasms. CMR can be used to differentiate between the two, diffuse interstitial or mid-wall fibrosis with non-coronary distribution suggesting an inflammatory process. In addition, a correlation has been found between myocardial fibrosis, skin thickness measurements and frequency of ventricular extrasystoles upon Holter monitoring, as well as an increased incidence in patients with early onset-disease, positive anti-neutrophil cytoplasmic antibodies (ANCA), pericarditis and myositis [20].

Among patients affected by inflammatory myopathies, myocarditis represents the most common cardiac pathology (38% of biopsy-proven cases). Cardiac involvement more frequently develops in patients having long-standing high activity of the disease. Screening of cardiac biomarkers, ECG and EchoCG is recommended for patients with polymyositis and dermatomyositis. Elevated levels of troponin T may correlate with disease activity but troponin I is more specific for detection of cardiac involvement. Non-specific changes in ECG are common and most patients present with slight changes in left ventricular ejection fraction upon EchoCG. Advanced cases show signs of diastolic dysfunction. CMR can display direct signs of myocardial inflammation and LGE for regional necrosis, therefore, it might be used for diagnosis in patients with known medical history and to monitor disease progression and treatment effectivity. However, positive CMR findings can persist after clinical remission has been achieved, increasing the risk of unnecessary prolonged treatment. The need for a biopsy is debatable [20,97,98]. 

In myasthenia gravis, myocarditis is rare (0.9–2.3%) but clinically severe. The risk of myocardial involvement is increased in patients with thymoma, of older age or female gender, or if the disease is caused by immune checkpoint inhibitors (ICI) such as programmed cell death protein 1 (PD-1) inhibitor. Additionally, myocarditis and myositis in myasthenia gravis patients can develop as a post-viral autoimmune syndrome, with presence of anti-muscle tissue antibodies, especially in patients with a thymoma. Clinical manifestations, ECG changes and CMR findings are non-specific but can be used for screening. Cardiac biomarkers and creatine kinase should be monitored in patients receiving ICIs. EchoCG can show spherical heart changes if performed during myasthenic crisis. EMB with a subsequent skeletal muscle biopsy is considered the method of choice to confirm myocarditis in the given clinical background [20,99,100,101,102].

The incidence of immune-mediated myocarditis in patients with inflammatory bowel disease is low (approximately 0.01%). Most of the cases and complications are associated with ulcerative colitis. The risk is increased in patients with a hypersensitivity reaction to 5-aminosalicylic acid or its derivates, used in the treatment of the primary disease. EMB can help to differentiate myocarditis from other entities, specify the prognosis or indicate if treatment corrections are needed [20].

Antiphospholipid antibody syndrome, eosinophilic granulomatosis with polyangiitis (known also as Churg–Strauss syndrome) and Takayasu arteritis are rare causes of immune-mediated myocarditis, usually with devastating consequences and high mortality. The diagnostic approach is limited by patients’ general condition. In most cases, the diagnosis is based upon medical history or confirmation of the primary disease by clinical findings and serology. Myocarditis is suspected clinically due to acute complications and confirmed via CMR or EMB, but occasionally revealed only in autopsy. Antiphospholipid antibody syndrome-caused myocarditis has a fulminant presentation with extensive deposits of immunoglobulin G, M, A and complement 3 fraction in myocardium. FDG PET/CT has been successfully used to determine ongoing myocardial inflammation and disease activity in Takayasu arteritis-associated cases. Eosinophilic granulomatosis with polyangiitis-associated myocarditis has been reported in patients with peripheral and extravascular eosinophilia, negative antineutrophil cytoplasmic antibodies (ANCA), history of severe bronchial asthma and findings of intracardiac thrombosis [1,20,103]. 

Kawasaki disease not only causes small and medium artery vasculitis but has an association with subclinical myocarditis that is proposed to be highly underdiagnosed. In its presence, mortality rates of the disease can reach 13.6%, mostly in children under 5 years of age. Spontaneous resolution is common, but long-term complications as fibrosis and myocyte degeneration have been reported, with EchoCG findings of systolic and diastolic dysfunction, needing further confirmation in follow-up studies. EMB is not considered necessary, as EchoCG screening is mandatory as soon as Kawasaki disease is suspected, with repeated follow-up EchoCGs and corrections of treatment regimen made according to findings [104]. 

### 3.4. Cardiotoxic Substance-Associated Myocarditis 

Although immune checkpoint inhibitors (ICI) represent a breakthrough achievement in oncology, these medications can cause adverse immunological events. Most of the side effects are mild and reversible, but in some cases (up to 8%), serious complications can result in considerably high mortality rates (36–67%). Myocarditis is the second most frequent of those, with a reported four-fold risk from ICI use. Concomitant myositis is reported in a high number of cases (up to 40%) [105,106,107,108]. Pooled incidence of adverse cardiac events is more common in dual treatment plans [109,110]. These events have been reported after detecting inflammatory infiltration in biopsy or autopsy samples, usually within 2–3 months after starting the treatment [111]. Autoantibodies (e.g., against cardiomyocytes or cardiac troponin T and myositis-associated antibodies) have been detected in cardiac muscle and blood samples, however, their significance is unknown due to a small number of reported cases. Common findings in biopsy materials were CD4+ and CD8+ lymphocyte infiltrates, showing a direct immune reaction towards an unknown target. In the result, fibrosis of the myocardium and the conduction system develops, frequently inducing conduction disorders [112,113,114,115]. Baseline ECG, cardiac troponin I and creatine kinase, with weekly check-ups for the initial 6 weeks of treatment have been suggested for monitoring and patient selection for a biopsy [1,116,117]. 

Review of cardiotoxic effects of biotherapy and molecular targeted therapy in treatment of neuroendocrine neoplasms with somatostatin analogues, tryptophan hydroxylase, mTOR and tyrosine kinase inhibitors found that biotherapy has a significantly lower incidence of adverse cardiac events and exacerbations of pre-existing conditions. EMB confirmed myocarditis has been reported in only one patient so far [118].

Among other reviews on medication-caused myocarditis, clozapine-associated cardiac adverse reactions have been evaluated. The rate of myocarditis was low (less than 1%), with a high chance of diagnostic errors as most cases were diagnosed only by symptoms, elevated levels of cardiac biomarkers, and changes in ECG or EchoCG. CMR and EMB were rarely applied for confirmation. Moreover, a reaction towards withdrawal and reintroduction of the medication at fault was not described [119,120,121]. EMB might be contraindicated in patients presenting with acute psychosis, however, false diagnosis of myocarditis could cause unnecessary discontinuation of the antipsychotic treatment [120]. In several studies, clinical symptoms were reported within the first 3–4 weeks of treatment, highlighting the relevant period for screening and biopsy acquiree [119,122,123].

Use of illegal substances such as psychomotor stimulants, especially in combination with alcohol, is known to have detrimental effects upon the cardiovascular system. Reactive oxygen species, toxic metabolites and direct activity on sodium and potassium ion channels, peripheral resistance, conductivity and myocardial contractility can induce exacerbations of pre-existing conditions, acute adverse events and even myocarditis [124,125,126]. Myocarditis has been reported as a hypersensitivity reaction with mononuclear cell infiltration and necrosis, which was not dependent on the dose or duration of stimulant use. In chronic users, scattered necrosis, loss of myofibrils, myocyte degeneration and edema were found in autopsy. This knowledge and acquiree of biopsy can be helpful in cases of unclear etiology or when use of illegal substances is denied [124]. 

Phospholipase A2 has been identified as the main cardiotoxic substance carried by venomous snakes. It can cause direct cardiomyocyte damage and a hypersensitivity reaction, resulting in a rare occurrence of myocarditis detectable in biopsy samples [127].

### 3.5. Specific Histopathological Subtypes of Myocarditis 

#### 3.5.1. Eosinophilic Myocarditis 

Myocardial inflammation due to infiltration of predominantly eosinophilic leukocytes has been reported in eosinophilic granulomatosis with polyangiitis (known also as Churg–Strauss syndrome), hypereosinophilic syndrome, parasitic infections, hypersensitivity reactions, specific types of malignancies and as idiopathic findings [1,128]. 

Three stages have been described in hypereosinophilic syndrome, which can be seen in other associated conditions, with varying severity. In the initial stage, mature eosinophils infiltrate the myocardium, causing inflammation with detectable necrosis. Hypereosinophilia or atypical lymphocytosis can be present within peripheral blood samples. History of fever and skin rash is commonly noted. The following stage is characterized by valvular involvement, apical obliteration and intracardiac thrombosis. The last stage represents chronic inflammation, endomyocardial fibrosis and formation of restrictive cardiomyopathy, with a progression rate in untreated cases as high as 90%. CMR can be used to detect intracardiac thrombosis and subendocardial fibrosis with LGE, both typical for eosinophilic myocarditis. Myocardial mapping can be used for further differentiation, decreasing the need for a biopsy. Notably, in Churg–Strauss syndrome, EMB can lack the typical morphological picture of perivascular and extravascular palisading granulomas (i.e., central eosinophilic necrosis surrounded by palisading giant cells) in association with necrotizing small vessel vasculitis, featuring fibrinoid necrosis and ruptured internal elastic lamina. Such histological presentation is attributable to the limited size of the tissue sample. Extravascular eosinophilic infiltrates and degranulation of eosinophils can remain the only hints towards the correct diagnosis that should be substantiated by complex evaluation of clinical and morphological criteria [1,17,93,103,129]. 

Symptoms, EchoCG, CMR findings and confirmation of associated conditions can be used for patient selection for a guided treatment approach and prognostic evaluation, without performing a biopsy [130]. For example, hypersensitivity reactions to drugs are more frequent in females (61.9%). Several organs are usually involved (83% of cases), mostly affecting the liver and kidneys. Cardiac lesions are common (29%), usually presenting in a delayed manner. Allopurinol and minocycline have been frequently reported as causes (12% and 19%, respectively) [1,17,131]. Reports of parasitic infections mention malaria, schistosomiasis, filariasis and helminths. Autoantibodies against myocardial proteins (e.g., anti-myosin) and high levels of inflammatory cytokines can be detected. Multifactorial predisposition is suspected, including genetic factors, dietary deficits and intake of eosinophil-stimulating substances (e.g., cyanogenic glucosides) [132,133,134,135].

#### 3.5.2. Giant Cell Myocarditis

Giant cell myocarditis (GCM) is a rare subtype of myocarditis, known to get mistaken with cardiac sarcoidosis and lymphocytic myocarditis, if appropriate diagnosis is not made by EMB, showing pathognomonic findings of diffuse necrosis and multinucleated giant cell infiltration. This can be detrimental considering that GCM has a more severe clinical presentation, needs cyclosporin-based treatment which is not used routinely and has an overall worse prognosis, with rates of transplantation and mortality reaching 80–100% in undiagnosed patients [1,136,137]. Survival probability is significantly increased, and the relapse rate can be controlled if adequate immunosuppression is started before mechanical circulatory support. Still, often (25.6%) the definitive diagnosis is made after tissue sampling during implantation of a left ventricular assist device or heart transplantation [138]. A helpful criterion for choosing to perform a biopsy would be the severity of GCM-caused clinical symptoms, coinciding with two of the main recommendations for performing an EMB in the first place [1,6,139,140]. 

#### 3.5.3. Myocardial Tuberculosis

*Mycobacterium tuberculosis* is the leading infectious cause of lethal outcomes worldwide, especially in patients with concomitant immunocompromising diseases. Cardiovascular involvement, mainly involving the pericardial sack, is one of the most common extrapulmonary manifestations and significantly worsens prognosis. Myocarditis is a relatively rare (less than 2% of cardiovascular involvement) but severe cardiac manifestation, more frequent in immunocompromised patients affected by miliary tuberculosis or hematogenous spread. The diagnostic pathway includes ECG, chest X-ray and EchoCG findings with a following CMR in cases of systolic, diastolic or contractility dysfunction upon EchoCG. Pericardial effusion is frequent, therefore confirmation of the infection within pericardiocentesis samples can avert the need for EMB for a definitive diagnosis [141].

#### 3.5.4. Lyme Carditis

*Borrelia burgdorferi*-caused cardiac complications can occur long after the initial infection from a tick bite. Approximately 10% of Lyme disease patients develop cardiac lesions, mainly (90%) Lyme carditis, presenting with a high-degree atrioventricular block. Suspicion index (Table 4) for risk stratification has been proposed, which could be used to select patients for further diagnostics. The reported sensitivity exceeds 93%, therefore biopsy precedence remains low [142,143].

#### 3.5.5. Chronic Inflammatory Cardiomyopathy

**Figure 4:**
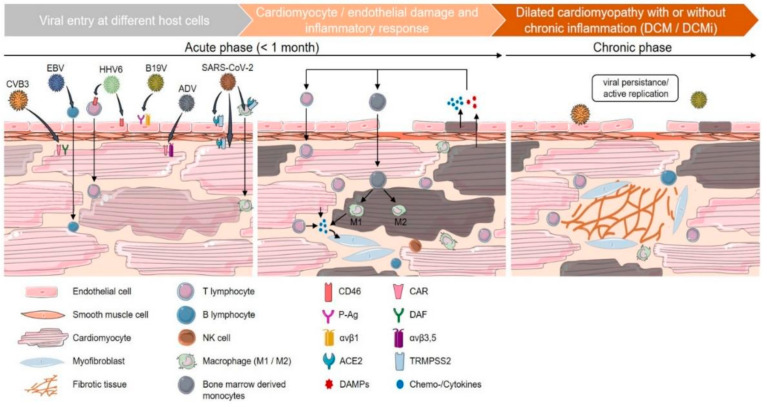
Chronic inflammatory cardiomyopathy: Acute phase showing entry routes of different pathogens—coxsackievirus B3 (CVB3), Epstein–Barr virus (EBV), human herpesvirus 6 (HHV6), parvovirus B19 (B19V), adenovirus (ADV), severe acute respiratory syndrome coronavirus 2 (SARS-CoV-2), through cell surface receptors or inflammatory cells, resulting in direct and indirect cardiomyocyte damage. This is followed by cytokine and damage-associated molecular pattern (DAMP) release, triggering infiltration with T-lymphocytes and monocytes, which differentiate into M1 or M2 macrophages. Inflammation-promoted cytokine release leads to activation of myofibroblasts; Chronic phase showing viral and/or inflammatory process persistence contributing to loss of cardiomyocytes and production of fibrotic tissue, which results in cardiomyopathy. Figure replicated from [23] under Creative Commons license, provided at https://creativecommons.org/licenses/by-nc/4.0/ (accessed on 1 February 2022). Changes made: Figure legend.

Chronic myocarditis is described as an intermediate state for patients that have signs of active myocarditis progressing towards the development of inflammatory cardiomyopathy, mainly as an immune-mediated injury with no evidence of a certain pathogen or cardiotoxic substance. The suggested definition describes a persistent inflammatory condition with symptom onset more than a month before the biopsy, where myocyte diameter abnormalities, fibrosis and scarce inflammatory infiltrates are found, with no evidence of necrosis (Figure 4) [1,144,145]. 

Length of symptoms exceeding a month, persistently elevated cardiac biomarkers, dilatation or hypokinesis of unknown origin seen upon EchoCG, unresponsiveness to standard heart failure treatment, CMR findings and detection or history of slowly progressing infections and known autoimmune disorders can be used as grounds for patient selection to perform an EMB. Genetic predisposition has been proposed, therefore specific medical history, positive family history or geographical factors could be taken into consideration. Recently, complementary evaluation of microRNAs has been suggested, yielding conflicting results that need further confirmation. The grade and type of infiltration in biopsy samples, as well as the triggering pathogen can be used to estimate the prognosis of mortality and transplantation necessity [1,23,144,145,146,147,148,149,150,151,152].

#### 3.5.6. Arrhythmogenic Cardiomyopathy

Arrhythmogenic cardiomyopathy is defined by the Heart Rhythm Society as a myocardial disorder that can be caused by a wide spectrum of genetic, systemic, infectious and inflammatory disorders. Initial life-threatening arrhythmogenic presentation of myocardial injury can be seen with undetected cardiac amyloidosis, sarcoidosis, Chagas disease, myocarditis and congenital cardiomyocyte protein disorders. Repeated exacerbations or subclinical inflammatory progression can result in fibrofatty replacement of normal myocardial tissue, serving as a substrate for rhythm disturbances. Diagnosis of an inherited disorder does not rely on a single gold standard test, but is achieved through a scoring system of familial, genetic, ECG, EchoCG and CMR results. EMB is reserved for patients in whom final diagnosis is unclear without histopathological exclusion of sarcoidosis, dilated cardiomyopathy or myocarditis. CMR analysis and electroanatomic voltage mapping is advised for sampling site precision, as typical sampling sites are not commonly affected [91,149,153,154,155,156,157,158].

#### 3.5.7. Myocardial Calcification

Myocardial calcification is a rare and life-threatening complication of myocarditis, ischemic heart disease, cardiac surgery, rheumatic fever or sepsis. This entity is pathogenetically mediated by the inflammatory and oxidative stress, which causes injury of cardiomyocytes and consequent dystrophic calcification in patients with normal calcium homeostasis. Calcification mostly affects the left ventricle within its outer layers. These changes are evident upon plain chest radiography, EchoCG, chest computed tomography (CT) and CMR. The best evaluation quality and precision can be reached without EMB, by chest CT, which might be indicated by clinical symptoms, a chest X-ray showing an increased density of the heart outline and/or large deposits or an EchoCG detecting increased echo-density and acoustic shadowing of the myocardium, along with hypokinesia. If CT is contraindicated, CMR can reveal extensive LGE and pericardial effusion [159].

## 4. Discussion

EMB holds a critical role for treatment correction in specific histological subtypes of myocarditis [1,137,155], differentiation of immune-mediated myocarditis from secondary infections, linked to immunosuppressive treatment [20,89,90,91,92,93,94,95,96,97,98,99,100,101,102,103,104] and explanation of unresponsiveness to conventional heart failure treatment [23,144]. To increase the diagnostic quality of EMB, the time period of an initial two weeks of symptom presence, together with a synergy of imaging methods, such as EchoCG with two-dimensional speckle tracking, CMR and myocardial mapping are advised [18,19,20,22]. Low application of EMB is either left unstressed in most studies or related to safety concerns, even despite evidence of low risk of complications in adult patients, when performed by experienced professionals [4,20]. This avoidance to perform the manipulation could cause a low number of high qualified professionals in the future. Scarcity of available data in children is continuously noted, and the existing data point out higher procedure-related complication rates than in adults [19]. The current recommendations for performing an EMB (Table 2), essentially, leave the choice to perform a biopsy in other patients within the hands of the attending clinician. 

Technology advancements of CMR and studies of its application have shown a considerable rise in its diagnostic accuracy when used alone [4,12,13], a predictive capacity of the method in patients surviving myocarditis [14,15,16] and potential of improvement. Furthermore, CMR can detect findings typical for eosinophilic myocarditis—intracardiac thrombosis and subendocardial fibrosis, and myocardial mapping can be used for further differentiation [1,17,93,129], introducing an alternative for diagnosis of one of the specific subtypes of myocarditis.

Viral infections are the most common reported etiology of myocarditis, presumed even in cases where the pathogen cannot be detected or when it could act as a trigger. No consensus exists regarding exclusion of infectious causes [4,21,24,25], needing development and confirmation of an initial algorithmic approach from experts of the field, promoting EMB use in ambiguous cases. Viruses have different patterns of myocardial involvement, actualizing the need for use of unified terminology, which should clearly distinguish between virus-mediated and virus-triggered myocarditis [1]. Several serum cardiac autoantibodies and circulating microRNAs have been detected as possible markers for differentiation [4,17,23,31,33,34]. Sufficient cardio-protection can be achieved with knowledge of pathophysiological patterns of different cardiotropic viruses, together with medical history, infection markers, viral load measurements and early detection of myocardial inflammation via non-invasive investigations, such as EchoCG and CMR, guiding the treatment plan and alleviating or emphasizing the need for a biopsy [6,25,26,35]. Furthermore, it is believed that delayed EMB has a low diagnostic value regarding the etiology [21,31], calling for specific recommendations regarding the time period within which a biopsy should be obtained. On the other hand, without a sufficient number of tissue samples confirming similarities or specific nuances of different etiology viral myocarditis, this knowledge of pathophysiological patterns cannot be obtained.

Support for empirical treatment without EMB confirmation of etiology and conventional heart failure treatment recommendations from the European Society of Cardiology [2,3] was seen in findings where immunosuppressive therapy in unspecified myocarditis show either positive or neutral effects in adults. PCR findings have no effect upon prognosis, and significant reduction of fibrosis is seen from conventional heart failure treatment alone [6,15,35,39]. Still, the listed data support indications for EMB in patients that lack a response to empirical treatment. Further studies are necessary in children, as conflicting results have been reported—fever adverse events with limited immunosuppression in cases of confirmed viral myocarditis [19,36], but a benefit from immunosuppression in clinically suspected myocarditis [37,38]. 

In COVID-19 patients, high probability of misdiagnosing myocarditis was reflected, as a large part (up to 48%) of COVID-19 infection-associated myocarditis cases were confirmed with only EchoCG findings [52,53,54,55], CMR findings non-compliant to Lake Louise criteria [56] or EMB findings not meeting Dallas criteria [57,61,62]. Objective findings highlight the hypothesis of a virus-triggered hyperinflammatory response [63,64,65,66,67,68]. This assumption is complemented by a high incidence of clinically suspected myocarditis in pediatric multisystem inflammatory syndrome [50,70,71,72,73] and autopsy findings of cardiac injury being a consequence of imbalanced immune response, hypercoagulation and hyperperfusion, pre-existing conditions or multiorgan failure [61,63,64,65,66,67,68,69]. In addition, COVID-19 vaccine safety reviews had low rates of CMR and EMB confirmation [17,84,85,86,87], causing difficulties to determine whether these reactions meet myocarditis criteria. In the light of the COVID-19 pandemic, EMB and autopsy sample collection is of utmost importance to identify the underlying pathogenetic process and possible treatment directions, when facing novel pathogens [76,77].

Evidence of systemic immune-mediated disease-associated myocarditis shows that clinical features of the primary condition are non-reliable in the diagnostics of myocarditis, as considerably high rates of isolated cardiac involvement have been reported [20,89,90,91,92,93,94,95,96]. Generally, myocarditis was clinically suspected through a synergy of medical history, serology, EchoCG, CMR, and PET, in most cases significantly deteriorating the prognosis for the pre-existing condition [20,89,90,91,92,93,94,95,96,97,98,99,100,101,102,103,104], so high clinical vigilance for cardiac involvement in the according patient group should be maintained. EMB, outside from patients with severe clinical presentation, should be performed to differentiate immune-mediated myocarditis from secondary infections, linked to immunosuppressive treatment.

Cardiotoxic drugs are widely used in treatment of oncological and psychiatric conditions, with recommended adverse effect monitoring periods and tools described for specific medications. ICI treatment has a confirmed ability to induce autoimmune myocarditis; autoantibodies of unknown significance are detected, representing a future research target [105,106,107,108,109,110,111,112,113,114,115,116,117]. Clozapine-induced myocarditis is mainly identified by non-specific findings or symptoms. Such an approach involves a high likelihood of diagnostic errors and risk for unnecessary withdrawal of the medication [119,120,121,122,123]. However, there are understandable difficulties to obtain patient consent or cooperation for CMR or EMB, due to the underlying health condition. Overall, EMB in substance-related events is necessary for scientific purposes, to evaluate pathogenesis, possible treatment options and correct determination of adverse events.

The use of EMB in different subtypes of myocarditis is summarized in Table 5. 

## 5. Conclusions

Endomyocardial biopsy, if performed properly and with the use of adjunct diagnostic methods, holds a critical role for treatment correction in specific histological subtypes of myocarditis, differentiation of immune-mediated myocarditis from secondary infections, linked to immunosuppressive treatment and explanation of failure to respond to conventional heart failure treatment. Significant problems in misdiagnosing have surfaced, asking for a careful compliance with diagnostic criteria and use of terminology within the international medical society. Further research in the children population is immensely needed. There is a high variety of possible focus points for future studies, from diagnostic properties of microRNAs and autoantibodies to follow-up studies.

## Figures and Tables

**Table 1:** Lake Louise criteria for patients with suspected myocarditis [1,2].

**Original Lake Louise Criteria ^1^**
Hyperemia shown by early gadolinium enhancement/T1-weighted images
Myocardial edema shown by increased relaxation time/intense T2-weighted images
Non-ischemic necrosis/fibrosis shown by late gadolinium enhancement (LGE)
**Updated Lake Louise Criteria ^2^**
T1-based imaging: increased native T1 or extracellular volume, non-ischemic LGE T2-based imaging: increased native T2, high signal intensity ratio
**Supportive Criteria**
Regional or global left ventricular hypokinesia Pericardial effusion

^1^ Confirmed 2 out of 3, have a reported 74% sensitivity and 86% specificity for definitive diagnosis. ^2^ Confirmed 2 out of 2, have a reported increase in sensitivity and specificity (87.5% and 96.2%).

**Table 2:** Recommended indications for endomyocardial biopsy [1,3].

**Main Indications in Patients with Clinical Suspicion of Myocarditis ^1^**
New-onset (2 weeks–3 months) heart failure with hemodynamic compromise, life-threatening ventricular arrhythmias or high-degree atrioventricular block Lack of short term (<2 weeks) response to standard medical treatment
**Additional Considerations in Patients with Clinical Suspicion of Myocarditis ^1^**
Patients receiving cardiotoxic medications Patients with known autoimmune disorders

^1^ Clinical presentation of chest pain, dyspnea, left/right-sided heart failure, arrhythmia or sudden cardiac arrest, with more than one of mandatory diagnostic tests (complete blood count, cardiac biomarkers, ECG, EchoCG, CMR) showing changes that are not caused by coronary artery, valvular and congenital heart disease or other known causes.

**Table 3:** Updated Dallas criteria for analysis of biopsy samples in suspected myocarditis [1,2].

**Histological Criteria of Active Myocarditis**
Myocardial infiltration with mainly mononuclear cells, signs of non-ischemic myocyte necrosis, with or without fibrosis, at routine light microscopy
**Immunohistochemical Criteria**
Infiltrate containing ≥14 leukocytes/mm^2^, including up to 4 monocytes/mm^2^ and CD3+ T-lymphocytes ≥ 7 cells/mm^2^

Abbreviation in the table: CD, cluster of differentiation.

**Table 4:** Lyme carditis suspicion index [142].

Factor	Points ^1^
*Erythema migrans*	4
History of tick bite	3
Constitutional symptoms of the infection ^2^	2
Endemic area inhabitant status	1
Male gender	1
Age under 50 years	1

^1^ The score of 0–2 points indicates low suspicion; 3–6, intermediate and 7–12, high. ^2^ Fever, malaise, arthralgia, dyspnea.

**Table 5:** Summary of literature-based findings in specific settings of myocarditis.

**Viral Myocarditis**		
Unspecified etiology	[1,15,21,25,26,40,41,42,43]	EMB should be acquired in patients with a severe clinical course for treatment correction purposes. EMB with an adjunct PCR is optional to distinguishing between virus-mediated and virus-triggered myocarditis and for ambiguous cases.
COVID-19 associated	[56,57,58,59,60,76,77,78,79,80,81,82]	A rise in cardiac troponin levels in absence of other parameters, suggesting a severe course of the infection can be used as a criterion for patient selection for CMR and EMB. EMB and autopsy sample analysis is highly desirable when facing novel pathogens. Follow-up studies with CMR and EMB are desired to determine the specific features and possible consequences of persistent myocardial inflammation after COVID-19 infection.
**Systemic Immune-Mediated Disease Associated Myocarditis**		
Sarcoidosis	[20,30,78,89,91]	Use CMR for screening in patients with extra cardiac sarcoidosis, FDG PET/CT for EMB site precision if isolated cardiac sarcoidosis is suspected.
Systemic lupus erythematosus	[20,30,96]	Use SLEDAI for risk assessment in patients with known disease, CMR or FDG PET/CT for screening, EMB to differentiate if infectious etiology is suspected in immunosupressed patients.
Systemic sclerosis	[20]	Use CMR for screening in patients with internal organ fibrosis or diffuse scleroderma, differentiate from Raynauds phenomenon, EMB is optional.
Inflammatory myopathies	[20,97,98]	Use cardiac troponin I and EchoCG for screening, especially in patients with high disease activity, CMR for confirmation, EMB is optional.
Kawasaki disease	[104]	Use of EchoCG monitoring is mandatory, EMB for confirmation is considered unnecessary.
Others (myasthenia gravis, inflammatory bowel disease, antiphospholipid antibody syndrome, eosinophilic granulomatosis with polyangiitis, Takayasu arteritis)	[1,20,99,102]	EMB with a subsequent skeletal muscle biopsy should be performed in patients with suspicion of myasthenia gravis. Other disorders have a rare occurance with a severe presentation, approach is limited by patients’ general condition and can be based upon medical history or confirmation of the primary disease by clinical findings and serology if hemodinamic stability for EMB cannot be achieved.
**Cardiotoxic Substance-Associated Myocarditis**		
Immune checkpoint inhibitors, clozapine, biotherapy and molecular targeted therapy, illegal substances, snake venom.	[116,117,118,119,120,121,122,123,124]	Monitor biomarkers, ECG and EchoCG starting treatment with cardiotoxic drugs, confirm reactions with CMR or EMB. EMB can be used to differentiate use of illegal substances from other entities.
**Specific Histopathological Subtypes**		
Eosinophilic Myocarditis	[1,17,93,129]	Use CMR to detect intracardiac thrombosis and subendocardial fibrosis with LGE, both typical for eosinophilic myocarditis. Myocardial mapping can be used for further differentiation, EMB is optional.
Giant Cell Myocarditis	[1,20,139,140]	EMB is mandatory for correct treatment choice, characteristically severe presentation may be used as an indication.
Myocardial Tuberculosis	[141]	Use ECG, chest X-ray, EchoCG and CMR in cases of systolic, diastolic or contractility dysfunction. Pericardiocentesis can be used for confirmation.
Lyme Carditis	[142,143]	Suspicion index for risk stratification is sufficient to start empirical treatment.
Chronic Inflammatory Cardiomyopathy	[1,23,144,145,146,147,148,149,150,151,152]	Length of symptoms > 1 month, biomarkers, EchoCG findings, unresponsiveness to standard heart failure treatment, CMR findings and history of slowly progressing infections or autoimmune disorders can be used for patient selection to perform an EMB.
Arrhythmogenic Cardiomyopathy	[30,149,153,154,155,156,157,158]	Use a scoring system of familial, genetic, ECG, EchoCG and CMR results to suspect inherited disorders. EMB is reserved for exclusion of sarcoidosis, dilated cardiomyopathy or myocarditis. CMR analysis and electroanatomic voltage mapping is advised for sampling site precision.
Myocardial Calcification	[159]	Use chest X-rays and/or EchoCG for screening, confirm with chest CT, CMR if CT is contraindicated.

Abbreviations in the table: CMR, cardiac magnetic resonance; FDG PET/CT, ^18^F-fluorodeoxyglucose Positron Emission Tomography/Computed Tomography; EMB, endomyocardial biopsy; SLEDAI, Systemic lupus erythematosus disease activity index.

## Data Availability

Not applicable.
